# Phylogeography of a good Caribbean disperser: *Argiope argentata* (Araneae, Araneidae) and a new ‘cryptic’ species from Cuba

**DOI:** 10.3897/zookeys.625.8729

**Published:** 2016-10-19

**Authors:** Ingi Agnarsson, Stephanie M. LeQuier, Matjaž Kuntner, Ren-Chung Cheng, Jonathan A. Coddington, Greta Binford

**Affiliations:** 1 Department of Biology, University of Vermont, Burlington, VT, USA; 2 Department of Entomology, National Museum of Natural History, Smithsonian Institution, Washington, DC, USA; 3 Evolutionary Zoology Laboratory, Biological Institute ZRC SAZU, Ljubljana, Slovenia; 4 Department of Biology, Lewis and Clark College, Portland, OR, USA

**Keywords:** Biogeography, CarBio, dispersal, diversification, GAARlandia, Intermediate dispersal model, Isolation by distance model

## Abstract

The Caribbean islands harbor rich biodiversity with high levels of single island endemism. Stretches of ocean between islands represent significant barriers to gene-flow. Yet some native species are widespread, indicating dispersal across oceans, even in wingless organisms like spiders. *Argiope argentata* (Fabricius, 1775) is a large, charismatic, and widespread species of orb-weaving spider ranging from the United States to Argentina and is well known to balloon. Here we explore the phylogeography of *Argiope argentata* in the Caribbean as a part of the multi-lineage CarBio project, through mtDNA haplotype and multi-locus phylogenetic analyses. The history of the *Argiope argentata* lineage in the Caribbean goes back 3-5 million years and is characterized by multiple dispersal events and isolation-by-distance. We find a highly genetically distinct lineage on Cuba which we describe as *Argiope butchko* **sp. n.** While the *argentata* lineage seems to readily balloon shorter distances, stretches of ocean still act as filters for among-island gene-flow as evidenced by distinct haplotypes on the more isolated islands, high F^ST^ values, and strong correlation between intraspecific (but not interspecific) genetic and geographic distances. The new species described here is clearly genetically diagnosable, but morphologically cryptic, at least with reference to the genitalia that typically diagnose spider species. Our results are consistent with the intermediate dispersal model suggesting that good dispersers, such as our study species, limit the effect of oceanic barriers and thus diversification and endemism.

## Introduction

The Caribbean diversity hotspot has been colonized by a number of lineages via varying routes over millions of years. As is typical of other old oceanic islands, the archipelago’s isolation helped form numerous single-island endemic species (Agnarsson and Kuntner 2012; Gillespie and Roderick 2002; Ricklefs and Bermingham 2008; Warren et al. 2015). The Caribbean islands are diverse in origin. Some are Darwinian volcanic islands that have been colonized exclusively by overwater dispersal – airborne or across the ocean, e.g. via vegetation rafts. Others are Wallacean fragment islands whose periodic connection to the mainland may have facilitated colonization over land bridges such as GAARlandia (Iturralde-Vinent and MacPhee 1999; Ricklefs and Bermingham 2008). Regardless, all the Greater Antilles islands and most of the minor Antilles have been isolated for the last several million years (Ali 2012; Heinicke et al. 2007; Iturralde-Vinent and MacPhee 1999; Iturralde-Vinent 2006). Thus the processes of divergence and diversification among islands due to lack of gene-flow can be expected to be ongoing in all but the best dispersing organisms for which stretches of ocean do not present formidable barriers—one prediction of the intermediate dispersal model (IDM) (Agnarsson et al. 2014; Claramunt et al. 2012; Weeks and Claramunt 2014). Such organisms are typically flying animals, or plants with salt-tolerant floating seeds, that are widespread but species depauperate (Weeks and Claramunt 2014).

Being wingless, a relatively small proportion of arachnid lineages tend to colonize ocean islands. Single-island endemism is common in successfully colonizing lineages (Arnedo and Gillespie 2006; Arnedo et al. 2007; Gillespie 2005; Gillespie et al. 2008; Zhang and Maddison 2012), a pattern consistent across taxa, islands and archipelagos including the Caribbean (Alayon 2006; Cosgrove et al. 2016; Crews and Gillespie 2010; Dziki et al. 2015; Esposito et al. 2015; McHugh et al. 2014; Zhang and Maddison 2012). This pattern is also found in many other invertebrates, and in vertebrates and plants (Ricklefs and Birmingham 2008). However, this pattern is by no means universal and different lineages often show contrasting patterns, such as in certain species of the spider genus *Selenops* (Crews et al. 2010). Indeed, some spiders can readily disperse overwater by ‘ballooning’—becoming airborne on silk threads anchored to their spinnerets (Bell et al. 2005). For ballooning spiders stretches of ocean could be only partial barriers (filters) leading to predictions of limited diversification among islands. Our study subject here, *Argiope* spiders (Bell et al. 2005; Levi 1983), is potentially one such lineage.

Species of the genus *Argiope* are large, sexually dimorphic, charismatic spiders with brightly colored abdomens (Cheng and Kuntner 2014, 2015) that were noted by early taxonomists and among the first spiders to be described (Catalog 2015; Clerck 1757). Despite their large adult size, *Argiope* spiders are thought to be excellent dispersers because they occupy open tree-less habitats and have been documented to balloon (Bell et al. 2005). *Argiope argentata* (Fabricius, 1775) is a species ranging from the United States to the Caribbean islands and as far south as Argentina (Levi 1983). It occurs on practically every Caribbean island and is thus an interesting subject for phylogeographical studies on relatively good dispersers.

Here, we present mtDNA and morphological data on *Argiope argentata* collected throughout the Caribbean to reveal phylogeographical patterns within the Caribbean, to test the degree of genetic structure within and among islands, and to measure divergence in cases where genetic patterns reflect geography. We verify relationships among species with a multi-locus phylogenetic approach, and we also describe a new species, *Argiope butchko* sp. n., previously thought to represent Cuban populations of *Argiope argentata*.

## Methods

**Figure 1.**
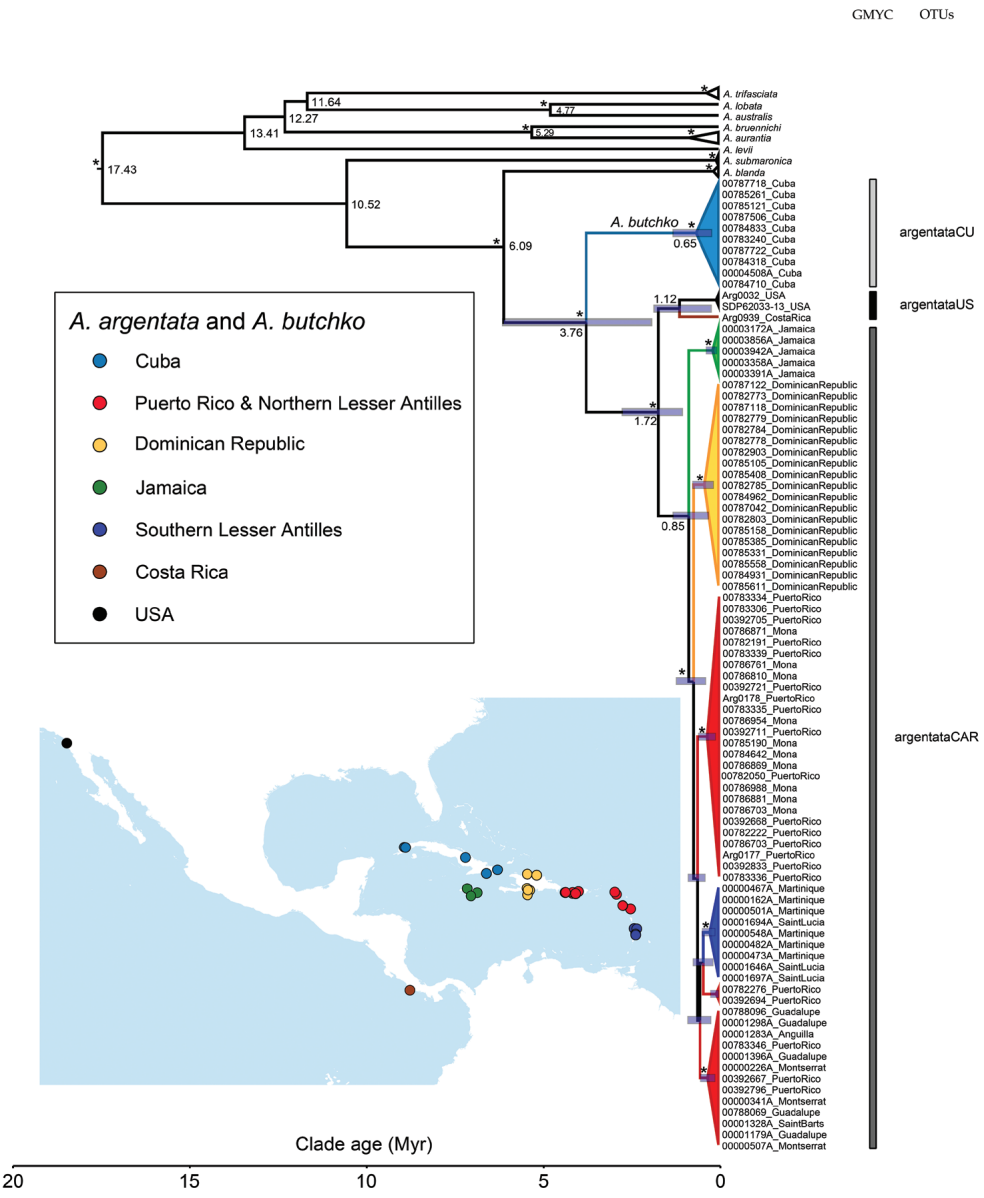
A dated phylogeny of *Argiope argentata* in the Caribbean, and other *Argiope* relatives. Shown are the results of tree based species delimitation analyses (GMYC method) on a BEAST phylogeny (node ages in million years) and the location of spiders used in this study (inset picture). Asterisk denotes posterior probability support >95%. The OTUs (operational taxonomic units) correspond to a cryptic species, *Argiope butchko* sp. n., from Cuba (argentataCU) and populations from other Caribbean islands (argentataCAR) plus mainland (argentataUS) treated as conspecific (*Argiope argentata*).

Specimens of *Argiope argentata* s. l. were collected diurnally using standard aerial searching and beating methods from 2011-2015 across the Caribbean and in SE USA (Fig. 1, Suppl. material 4), including at four sites in Cuba: Siboney in Santiago, Alejandro in Guantanamo, Sierra de Camaguey in Camaguey, and Viñales, Sierra de los Órganos, Pinar del Rio. Specimens were preserved in 95% ethanol in the field and stored at -20 °C until DNA extraction. Two sequences of mainland American *Argiope argentata* and seven sequences of outgroups, downloaded from Bold and GenBank, were included in the analyses (Suppl. material 4). As outgroups we included eight *Argiope* species, including the closest relatives of *Argiope argentata* based on a recent molecular phylogeny (Cheng and Kuntner 2014) (see Suppl. material 4).

DNA was isolated from 85 *Argiope argentata* s.l. and 13 other *Argiope* species with the QIAGEN DNeasy Tissue Kit (Qiagen, Inc., Valencia, CA), or using phenol extraction (Suppl. material 4). We sequenced a fragment of the mitochondrial ‘DNA barcode’ Cytochrome c oxidase subunit 1-COI, a useful marker at low taxonomic levels in spiders, to establish boundaries among species (Čandek and Kuntner 2015; Hebert et al. 2003). To amplify COI we used the primers LCO 1490 and HCO 2198 (Folmer et al. 1994). PCR conditions and sequencing protocols were described previously (Bloom et al. 2014; McHugh et al. 2014). Sequences were submitted to GenBank (see Suppl. material 4 for accession numbers). Sequences were assembled using Phred and Phrap (Green 2009; Green and Ewing 2002) via Chromaseq (Maddison and Maddison 2011a) in Mesquite 3.03 (Maddison and Maddison 2011b) with default parameters. The sequences were proofread and then aligned using the online EMBL-EBI MAFFT (Katoh 2013). COI nucleotide sequences were translated to amino acids to check for stop codons and to detect interspecifically consistent amino acid differences.

For Bayesian analyses, the GTR+I+G model was selected as the appropriate substitution model by the AIC criterion (Posada and Buckley 2004) in jModeltest 2.1.4 (Darriba et al. 2012). We employed a Bayesian approach to phylogenetic reconstruction implemented in MrBayes 3.1.2 (Ronquist et al. 2012). Two independent runs, each with four Markov chain Monte Carlo (MCMC) chains, were performed simultaneously with random starting trees, and the MCMC process was run for 10,000,000 generations, with a sampling frequency of 100 and a burn-in of the first 25% generations. We then ran BEAST (Drummond and Rambaut 2007; Drummond et al. 2012) for dating analyses of the mtDNA data. The BEAST run comprised 40,000,000 generations, using a lognormal relaxed clock with fixed estimated substitution rate (mean = 0.0112, SD = 0.001) (Bidegaray-Batista and Arnedo 2011), assuming a birth-death speciation model for the tree prior, with the best fit substitution models, and default options for all other prior and operator settings. The final consensus tree was produced in TreeAnnotator v1.8.0, with 25% burn-in.

To test the phylogenetic relationships from COI data, we also ran Bayesian analysis with a multi-locus dataset with two nuclear markers (28S and Histone 3) and 1–2 exemplars per species. The PCR reactions of 28S and Histone 3 followed established protocols for argiopine spiders (Cheng and Kuntner 2014). The Bayesian analysis was performed with the GTR+I+G model identified as the best fit substitution model for all loci, and using all other settings as above.

To test for cryptic species in *Argiope argentata*, we used a combination of tree-based species delimitation methods and genetic distances. For tree-based species delimitation method, the General Mixed Yule-Coalescent model with single threshold (GMYC) (Pons et al. 2006) was applied to the BEAST tree in R 3.0.3 (R Core Team 2014) with the Splits package (http://splits.r-forge.r-project.org/). We then calculated the genetic distance among potential OTUs as well as within and among the two species (with Cuban populations defined as putative species), and among all individual specimens. In all cases we used Kimura 2-parameter (K2P) (Kimura 1980) in Mega 6.06 (Tamura et al. 2013). (Table 1). Genetic distances were then correlated with geographic distances, the latter estimated (in m) from latitude and longitude data using the Geographic Distance Matrix Generator (Ersts 2016). Regression analyses between genetic and geographic distances were done in JMP Pro 11 and scatter plots produced in Excel and then modified in Illustrator. In addition to analyses including all ingroup individuals, regression analyses were run separately for intraspecific and interspecific comparisons to test the taxonomic hypothesis of *Argiope argentata* s. l. containing a cryptic Cuban species. The prediction here is that a correlation between genetic and geographic distances would hold within (e.g., Hamilton and Eckert 2007; Eckert et al. 2008), but not between, species as these should have non-geographic barriers to gene flow.

**Table 1.** Descriptive statistics for K2P (Kimura 2-parameter) distances within and between the molecular operational taxonomic units (OTUs), which were identified by molecular species delimitation methods.

Within OTUs			
OTU	N	K2P	
		Mean	Std. Err
argentataCAR	74	0.009	0.002
argentataUS	3	0.018	0.004
argentataCU	10	0.006	0.001
Between OUTs			
OTU 1	OTU 2	K2P	
		Mean	Std. Err
argentataCAR	argentataUS	0.029	0.006
argentataCAR	argentataCU	0.061	0.010
argentataUS	argentataCU	0.064	0.010

Fst and Kxy indexes were calculated in DNAsp v5 (Librado and Rozas 2009).

Haplotype networks were constructed using median-joining networks (Bandelt et al. 1999) in PopART (http://popart.otago.ac.nz/index.shtml) with default settings. Networks were exported as graphs and then edited in Adobe Illustrator.

Adult males and females were imaged using a Visionary Digital BK Plus digital imaging system. Specimens arranged in hand sanitizer and covered in 95% ethanol were photographed at dorsal, ventral, and lateral angles. Taxonomic measurements were derived from photographs in Adobe Photoshop. Genitalia observations and illustrations were made from photographs and by dissecting out the epigyna, digested in potassium hydroxide solution to remove soft tissue to make internal structures visible.

## Results

A fragment of COI (659 bp) was obtained for all individuals, and with added data from Genbank, making up a total of 107 sequences, including outgroups and 87 individuals morphologically identified as *Argiope argentata*. 540 base pairs overlapped for all individuals and missing data was 5.1%. Bayesian analyses of this dataset produced a topology that, with some internal node exceptions, was well supported (Figs 1, Suppl. material 1). This tree suggests that *Argiope argentata* s.l., being sister to *Argiope blanda*, contains a clade from Cuba and a clade that contains all other sampled populations. The phylogenetic structure within the latter suggests a grade of North American, Costa Rican, and the island Caribbean clades (not Cuba), respectively. Only Hispaniola and Jamaica have monophyletic island populations, and Martinique + St. Lucia together form a clade, other island populations do not emerge as monophyletic. To test the relationships between the major lineages suggested by mitochondrial-only results, we ran phylogenetic analyses of a subset of terminals with only nuclear data. The concatenated matrix consisted of 12 sequences (7 outgroups and 3 OTUs of *Argiope argentata*) and 1172 base pairs (28S - 829 bp and Histone 3 - 343 bp), with 1.3% missing data. These results (Suppl. material 2) confirm the core relationships among the Caribbean, North American, and Cuban populations of *Argiope argentata* s. l., respectively. Thus, both nuclear only and mitochondrial only phylogenies recover the sister relationship of Cuba with a clade that contains North American mainland plus other Caribbean island representatives. BEAST analyses, likewise, confirm these relationships (Suppl. material 3), the only significant difference being that Costa Rican and mainland American populations are monophyletic. Estimated node ages from BEAST, summarized in Fig. 1, date the mrca of *Argiope blanda* and *Argiope argentata* s.l. at roughly 6 Ma, and date the split between the Cuban clade and the remainder of *Argiope argentata* s.l. to about 3.8 Ma. The split between the mainland and Caribbean island populations of *Argiope argentata* s.s. is estimated at 1.7 Ma, and the Caribbean island ‘diversification’ is less than 1 Ma.

The GMYC analysis split the COI data into 11 OTUs, including eight correctly identified outgroup species. GMYC model provided a significantly better fit to the data than the null hypothesis of no structure (likelihood ratio: 27.06, *P* < 0.001), thus identifying 3 OTUs within *Argiope argentata* s. l. (Fig. 1): individuals from mainland C. and N. America (argentataUS), individuals from throughout most of the Caribbean (argentataCAR), and individuals from Cuba (argentataCU). The genetic distance test revealed very low K2P values within the OTUs (Table 1). In contrast, K2P values between the OTUs were all above 3%, but were particularly high between the Cuban OTU and the others (Table 1): While the Caribbean plus mainland OTUs comfortably fall within the intraspecific range typical for spiders, the average genetic distances between the Cuban OTU and the remaining two (average around 6%) were higher than the typical interspecific boundary in spiders (Čandek and Kuntner 2015).

**Figure 2.**
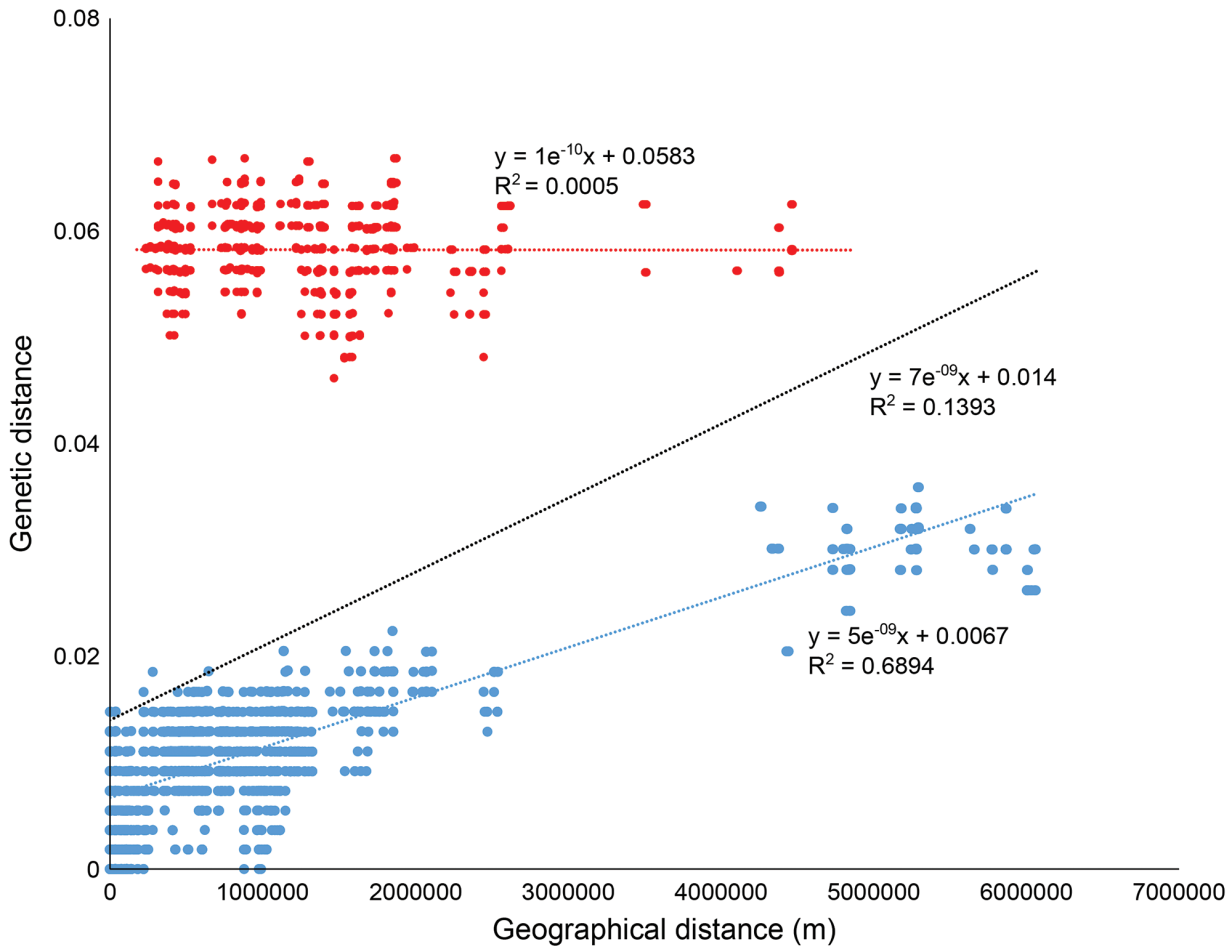
Regression analysis between geographic and genetic distances among all specimens of *Argiope argiope* s.l. included here. Blue dots and line represent within species comparisons and red dots and line those among the two species as here defined. Black line is regression across all data. Geographical distances well explain genetic distances within species, but not between the species, as expected.

Other measures of nucleotide differences (Kxy) and gene flow (F^ST^) likewise indicate particularly high distinction and genetic isolation of these lineages (Suppl. material 5, 6). Genetic and geographic distances were significantly correlated across the ingroup specimens (Fig. 2, R^2^=0.14, P<0.01). However, this correlation was entirely due to comparisons among specimens within each of the two species defined here (Fig. 2, R^2^=0.69, p<<0.01), whereas there was no correlation between genetic and geographic distances in comparison of specimens across species (Fig. 2, R^2^=0.0005, p>0.05). Thus, phylogenetic, population genetic, and species delimitation analyses all agree that the Cuban population is heterospecific with the broadly distributed *Argiope argentata*.

**Figure 3.**
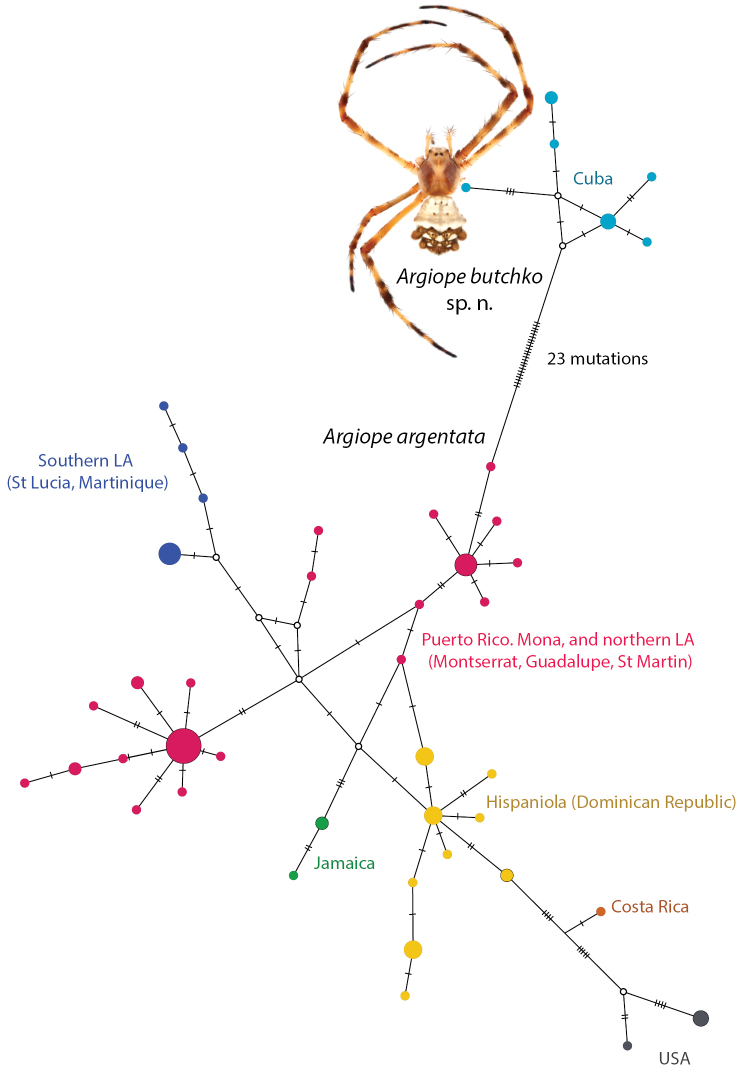
A haplotype network of Caribbean *Argiope argentata* and *Argiope butchko* sp. n. Haplotypes are colored by locality as indicated, circle size reflects number of individuals carrying that haplotype from 1-7 in total, open circles represent unobserved haplotypes. Hash marks indicate mutational differences among haplotypes. Inset photograph is of a female *Argiope butchko*.

Finally, haplotype analyses indicate clear phylogeographic structuring of COI haplotypes despite generally shallow divergences among islands (Fig. 3), as also indicated by the correlation between genetic and geographic distances (Fig. 2). More isolated islands have unique haplotypes, while haplotypes are shared among islands like Puerto Rico, Mona, and the northern Lesser Antilles. The Cuban population is a clear outlier in the haplotype network (Fig. 3), separated from other haplotypes by numerous mutations. Thus, based on all phylogenetic topologies and population genetic results we refer to the Cuban clade of *Argiope argentata* s. l. as *Argiope butchko* sp. n. and its sister clade as *Argiope argentata* s.s. (see Taxonomy for formal justification).

## Discussion

Archipelagos such as the Caribbean provide opportunities for colonization followed by isolation, restriction of gene flow, and the formation of local endemics (Ricklefs and Birmingham 2008). However, the degree of endemism will depend, in part, on the dispersal ability of the lineages in question (e.g. Claramunt et al. 2012; Diamond et al. 1976). We studied a spider lineage known to ‘balloon’ and thus expected to be able to cross oceanic barriers rather readily. We find that our sample of *Argiope argentata* s. l. from the Caribbean nevertheless represents several geographically structured populations and one highly genetically distinct lineage in Cuba (Figs 1, 3, Suppl. material 1–3). The larger Caribbean islands (Cuba, Hispaniola and Jamaica), and mainland continent, have genetically isolated populations (Fig. 3) among which there is likely limited or no recent gene-flow, while there seems to be movement and gene flow among islands in the eastern Caribbean. As we discuss below, isolation-by-distance (Fig. 2) coupled with a single speciation event, may readily explain these observed patterns.

We find strong evidence for two species in our dataset, the widespread *Argiope argentata* and one new species, *Argiope butchko* sp. n. from Cuba. Both genetic distances (Table 1), phylogenies, networks, and species delimitation analyses (Figs 1, 3, and supplementary material), and analyses of genetic vs geographic distances (Fig. 2) support this conclusion. Specimens from the Caribbean apart from Cuba are monophyletic with some island-level genetic structure (Fig. 3). Representatives from both Jamaica and the Dominican Republic are respectively monophyletic, with both islands sampled from widespread localities. However, patterns in the remaining Caribbean fauna are consistent with short-distance overwater dispersal, as evidenced by shared haplotypes between Puerto Rico and several of the Lesser Antilles east and south of it (Fig. 3). The relatively young age of these Caribbean lineages (3–5 my, Figs 1, S3) furthermore suggests that overwater dispersal has been the mode of colonization of each of the major islands. Thus, consistent with well documented ballooning behavior in *Argiope* (Bell et al. 2005), these spiders seem to be quite capable of crossing oceanic barriers. However, they do not seem to do so frequently enough to establish panmixia across the Caribbean. Therefore, processes of ongoing diversification result in island-specific haplotypes. That open ocean represents a filter rather than a barrier to dispersal is further evidenced by the strong correlation between geographic and genetic intraspecific distances (Fig. 2). Populations on nearby islands tend to be genetically close (or identical) with the greatest genetic distances, and splits exceeding 1.5 my, found between Caribbean island vs geographically distant mainland lineages. Such isolation-by-distance patterns are not unexpected when dispersal is restricted (e.g., Hamilton and Eckert 2007; Eckert et al. 2008) and have been found in other Caribbean taxa such as pines (Jardon-Barbolla et al. 2011). However, these patterns do not hold between the populations of *Argiope butchko* (Cuba) and the populations of *Argiope argentata* (from elsewhere). This is further evidence that *Argiope butchko* has undergone speciation after isolation for over 3 my (Fig. 1). The intermediate dispersal model (Agnarsson et al. 2014; Agnarsson and Kuntner 2012; Claramunt et al. 2012; Diamond et al. 1976) predicts that species richness across archipelagos peaks in intermediate dispersers but is comparatively low in excellent dispersers where oceanic barriers are less effective. Our findings are consistent with this model – *Argiope* spiders are effective dispersers and unlike most arachnid lineages studied in the Caribbean where single island endemism is prominent (Cosgrove et al. 2016; Crews and Gillespie 2010; Dziki et al. 2015; Esposito et al. 2015; McHugh et al. 2014) *Argiope* have undergone little diversification in the Caribbean. A similar pattern was found, for example, in some *Selonops* lineages in the Caribbean (Crews et al. 2010) and in the nephilids of the western Indian Ocean islands. In the latter group the excellent disperser *Nephila* showed relatively shallow intraspecific divergences among some islands but the poorer dispersers *Nephilingis* and *Clitaetra* have formed single island endemics (Kuntner and Agnarsson 2011a; b). High dispersal ability in *Argiope*, therefore, may have limited diversification in the Caribbean.

**Figure 4.**
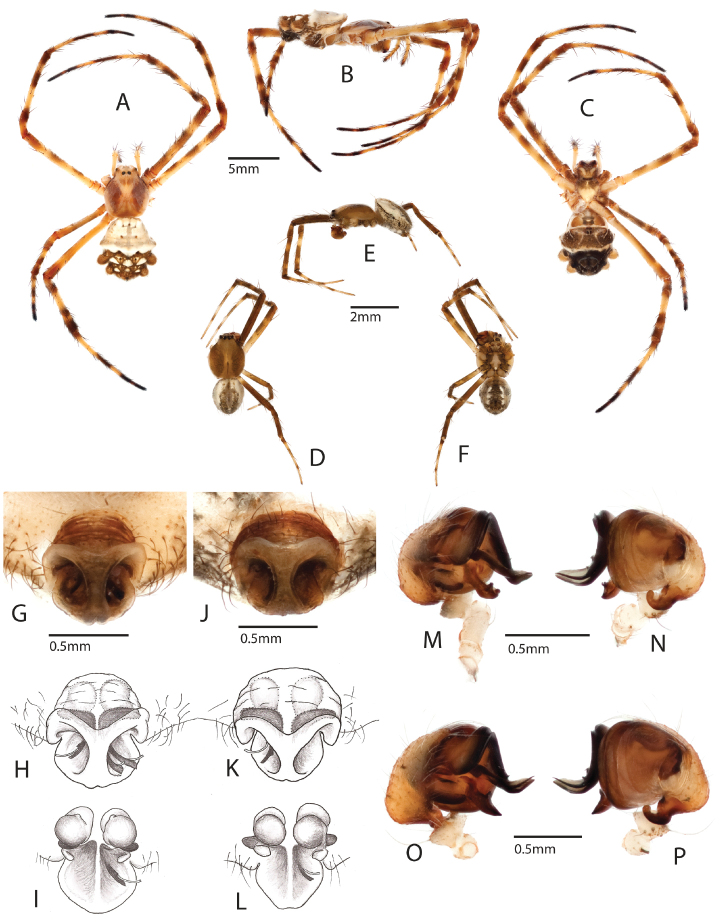
Female holotype *Argiope butchko* sp. n. **a** dorsal **b** lateral **c** ventral; Male paratype *Argiope butchko* sp. n. **d** dorsal **e** lateral **f** ventral **g** external epigynum **h** external epigynum illustration showing spermatheca and spiraling ducts **i** internal epigynum illustration dorsal **m** palp lateral **n** palp ventral; *Argiope argentata* **j** external epigynum **k** external epigynum illustration showing spermatheca and spiraling ducts **l** internal epigynum illustration dorsal **o** palp lateral **p** palp ventral.

**Figure 5.**
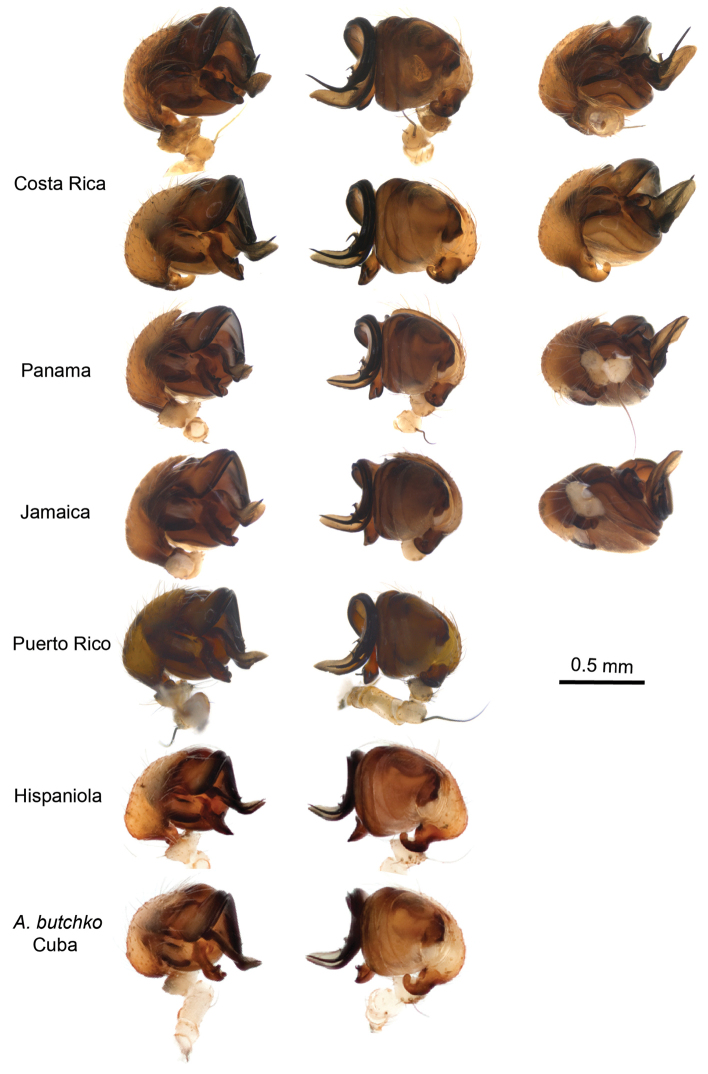
Comparative morphology of the male palpal organ of the widespread *Argiope argentata* and the new *Argiope butchko*. No clearly diagnostic features were identified in the new species, though slight differences in the terminal parts of the median apophysis and the embolus are observed and merit further comparative investigation.

Based on this finding we examined in detail the comparative morphology of *Argiope argentata* and the putative new species from Cuba, here described as *Argiope butchko* sp. n. We found no diagnostic differences in the morphology of male and female genitalia that would be consistent with the deep genetic divergence (~6.3% average sequence divergence, Table 1) and genetic isolation that has persisted for substantial time (Figs 1–2). Rather, variation seems profuse (Figs 4–5). Likewise, the Cuban species does not differ from *Argiope argentata* in body size measures. Hence, *Argiope butchko* sp. n. can be characterized as a morphologically ‘cryptic’ species. Further sampling and analysis of *Argiope argentata* DNA and morphology throughout its distribution range outside the Caribbean, especially in S. America, is a logical next step and may reveal additional species in this complex.

We note that our main results are based on a single mitochondrial locus and thus our phylogeographic conclusions are restricted to the picture expected from female inheritance. Nevertheless, multi-locus phylogenetic analyses support the general conclusions.

## Conclusions

Consistent with predictions of the intermediate dispersal model, our analyses of *Argiope argentata* mtDNA haplotype diversity and phylogeography across the Caribbean established that geographical structuring of haplotypes of this good disperser taxon was mostly consistent with a single widespread species model. Nevertheless, specimens from Cuba were deeply divergent from the remaining areas in all analyses, which we take as evidence for discovery of a cryptic species here described as *Argiope butchko* sp. n. ‘Hidden’ taxonomic diversity in the Caribbean is being revealed in multiple lineages by the CarBio project (Cosgrove et al. 2016; Dziki et al. 2015; Esposito et al. 2015; McHugh et al. 2014) and future work aims to test taxonomic hypotheses in other ‘widespread’ arachnid species that range from excellent to relatively poor dispersers and thus test the intermediate dispersal model at various taxonomic levels.

## Taxonomy

### Family Araneidae Clerk, 1757 Subfamily Argiopinae Simon, 1890 Genus *Argiope* Audouin, 1826

#### Argiope butchko

Taxon classificationAnimaliaAraneaeAraneidae

LeQuier & Agnarsson sp. n.

http://zoobank.org/CF438FAF-2E27-44A4-9FF3-1DADDDA942DD

##### Etymology.

The species epithet, a noun in apposition, honors the memory of Dennis Butchko, an inspiring science teacher.

##### Type material.

Female holotype from Siboney, Santiago de Cuba (19.9608°N, 75.7076°W), April 1, 2012, Col. Team CarBio, deposited in the Smithsonian (NMNH). Two female paratypes, one from the type location and one from Sierra de Camaguey, Camaguey, Cuba (21.5916°N, 77.7882°W). Three male paratypes, one from holotype location, one from Sierra de Camaguey, Camaguey, Cuba, and one from Viñales, Sierra de los Órganos ,Pinar del Rio, Cuba 22.6210°N, 83.7383°W. Paratypes will be deposited in the Smithsonian (NMNH).

##### Diagnosis.

*Argiope butchko* sp. n. differs from all other *Argiope* except *Argiope argentata* by the presence of the embolic distal curl (Levi, 2004: fig. 43, arrow). No distinct feature of the male palp and female epigynum were found that reliably diagnose Argiope butchko sp. n. from *Argiope argentata*.

*Argiope butchko* sp. n. and *Argiope argentata* can be diagnosed from one another, and other related *Argiope* species, on the basis of the following unique, synapomorphic, mtDNA nucleotide substitutions at the following standard DNA barcode alignment positions in each species (following Agnarsson et al. (2015)):

*Argiope butchko*: A (127), C (133), C (157) C (178), T (190), G (208), C (226), A (293), G (316), A (379, G (502), G (508), C (607); *Argiope argentata*: G (49), G (211), A (508), A (511), G (643).

##### Description.

Males and females of this species closely resemble *Argiope argentata* (Fabricius 1775; Levi 1983; Levi 2004). Males have a distal curl on the embolus (Levi 2004: fig. 43, arrow) and a median apophysis that is blunt at the tip (Figs 4m, o, 5). Females of *Argiope argentata* typically have a brown sternum with a median white line (Levi 1968). In *Argiope butchko* sp. n. the posterior half of the sternum is white or off-white and the anterior half is brown with a small median white dot on the anterior edge (Fig. 4c). However, this feature is not clearly diagnostic as variation is observed in *Argiope argentata*. The epigynum of *Argiope butchko* sp. n. has a wider posterior plate than Caribbean *Argiope argentata* and a smaller anterior bulge (Fig. 4G–I), but again variation in *Argiope argentata* outside the Caribbean suggests this feature is not diagnostic for the species.

*Dimensions* (mm). Holotype (female) - Total body length excluding chelicera 10.74, carapace length 5.00, carapace width 4.36. Leg I: femur length 8.28, patella and tibia length 8.63mm, metatarsus 8.00, tarsus 2.09. Leg II: patella and tibia length 8.06. Leg III: patella and tibia length 4.66. Leg IV: patella and tibia length 7.13.

*Variation* (mm). Female (N=4) - Total body length ranged from 10.29–10.74, carapace length 3.84–5.00, carapace width 3.16–4.36. Leg I: femur length 6.50–8.28, patella and tibia length 6.40–8.63, metatarsus 5.77–8.00, tarsus 1.80–2.09. Leg II: patella and tibia length 7.83–8.06. Leg III: patella and tibia length 3.73–4.66. Leg IV: patella and tibia length 4.66–7.13. *Male* (N=3) - Total body length ranged from 2.88–3.44, carapace length 1.70–1.88, carapace width 1.44–1.57. Leg I: femur length 1.62–2.14, patella and tibia length 1.99–2.37, metatarsus 1.74–1.88, tarsus 0.81–0.88. Leg II: patella and tibia length 1.41–1.90. Leg III: patella and tibia length N/A. Leg IV: patella and tibia length 1.40–1.63.

##### Distribution.

The species is restricted to Cuba.

##### Natural history note.

Three embolus tips were found embedded in the epigynum of a female *Argiope butchko*, one in the left opening and two in the right opening (Fig. 4g–h) This is similar to *Argiope argentata*, which has been known to have up to five embolic tips in one female (Jaeger 2012).

## Supplementary Material

XML Treatment for Argiope butchko

## Supplementary materials

Supplementary material 1 Figure S1 Data type: phylogeny data Explanation note: Bayesian phylogeny based on analysis in MrBayes using CO1 data This dataset is made available under the Open Database License (http://opendatacommons.org/licenses/odbl/1.0/). The Open Database License (ODbL) is a license agreement intended to allow users to freely share, modify, and use this Dataset while maintaining this same freedom for others, provided that the original source and author(s) are credited.Ingi Agnarsson, Stephanie M. LeQuier, Matjaž Kuntner, Ren-Chung Cheng, Jonathan A. Coddington, Greta Binford

Supplementary material 2 Figure S2 Data type: phylogeny data Explanation note: Bayesian phylogeny based on analysis in MrBayes using the two nuclear genes. This dataset is made available under the Open Database License (http://opendatacommons.org/licenses/odbl/1.0/). The Open Database License (ODbL) is a license agreement intended to allow users to freely share, modify, and use this Dataset while maintaining this same freedom for others, provided that the original source and author(s) are credited.Ingi Agnarsson, Stephanie M. LeQuier, Matjaž Kuntner, Ren-Chung Cheng, Jonathan A. Coddington, Greta Binford

Supplementary material 3 Figure S3 Data type: phylogeny data Explanation note: Detailed results from the BEAST analysis summarized in Fig. 1. This dataset is made available under the Open Database License (http://opendatacommons.org/licenses/odbl/1.0/). The Open Database License (ODbL) is a license agreement intended to allow users to freely share, modify, and use this Dataset while maintaining this same freedom for others, provided that the original source and author(s) are credited.Ingi Agnarsson, Stephanie M. LeQuier, Matjaž Kuntner, Ren-Chung Cheng, Jonathan A. Coddington, Greta Binford

Supplementary material 4 Table S1 Data type: specimen data Explanation note: Specimen data including the Genbank accession number. This dataset is made available under the Open Database License (http://opendatacommons.org/licenses/odbl/1.0/). The Open Database License (ODbL) is a license agreement intended to allow users to freely share, modify, and use this Dataset while maintaining this same freedom for others, provided that the original source and author(s) are credited.Ingi Agnarsson, Stephanie M. LeQuier, Matjaž Kuntner, Ren-Chung Cheng, Jonathan A. Coddington, Greta Binford

Supplementary material 5 Table S2 Data type: specimen data Explanation note: Results of Fst and Kxy (average number of nucleotide differences) analyses among specimens from all islands and mainland. The Cuban specimens, here described as a new species, stand out in both analyses indicating genetic isolation and divergence. This dataset is made available under the Open Database License (http://opendatacommons.org/licenses/odbl/1.0/). The Open Database License (ODbL) is a license agreement intended to allow users to freely share, modify, and use this Dataset while maintaining this same freedom for others, provided that the original source and author(s) are credited.Ingi Agnarsson, Stephanie M. LeQuier, Matjaž Kuntner, Ren-Chung Cheng, Jonathan A. Coddington, Greta Binford

Supplementary material 6 Table S3 Data type: specimen data Explanation note: Genetic and Geographic distances among all ingroup specimens used in generating Figure 2. This dataset is made available under the Open Database License (http://opendatacommons.org/licenses/odbl/1.0/). The Open Database License (ODbL) is a license agreement intended to allow users to freely share, modify, and use this Dataset while maintaining this same freedom for others, provided that the original source and author(s) are credited.Ingi Agnarsson, Stephanie M. LeQuier, Matjaž Kuntner, Ren-Chung Cheng, Jonathan A. Coddington, Greta Binford

## References

[B1] Audouin JV (1826) Explications sommaires des planches d’Arachnides de l’Egypte et de la Syrie. In: Savigny JC (Ed.) Description de l’Egypte et de la Syrie. Volume 4, 121.

[B2] Agnarsson I Cheng RC Kuntner M (2014) A Multi-Clade Test Supports the Intermediate Dispersal Model of Biogeography. PLoS ONE 9(1): . doi: 10.1371/journal.pone.0086780 10.1371/journal.pone.0086780 PMC3897756 24466238

[B3] Agnarsson I Jencik BB Veve GM Hanitriniaina S Agostini D Goh SP Pruitt J Kuntner M (2015) Systematics of the Madagascar Anelosimus spiders: remarkable local richness and endemism, and dual colonization from the Americas. ZooKeys 509: 13–52. doi: 10.3897/zookeys.509.8897 10.3897/zookeys.509.8897 PMC4493342 26175602

[B4] Agnarsson I Kuntner M (2012) The generation of a biodiversity hotspot: biogeography and phylogeography of the western Indian Ocean islands. In: Anamthawat-Jonsson K (Ed.) Current Topics in Phylogenetics and Phylogeography of Terrestrial and Aquatic Systems. Tech Publishers, Rijeka, 33–82. doi: 10.5772/38958

[B5] Alayon G (2006) Endemicidad y relaciones de las aranas (Araneae) de las Antillas Mayores. Cocuyo 16: 63–68.

[B6] Ali JR (2012) Colonizing the Caribbean: is the GAARlandia land-bridge hypothesis gaining a foothold? Journal of Biogeography 39: 431–433. doi: 10.1111/j.1365-2699.2011.02674.x

[B7] Arnedo MA Gillespie RG (2006) Species diversification patterns in the Polynesian jumping spider genus *Havaika* Proszynski, 2001 (Araneae, Salticidae). Molecular Phylogenetics and Evolution 41: 472–495. doi: 10.1016/j.ympev.2006.05.012 16837219 10.1016/j.ympev.2006.05.012

[B8] Arnedo MA Oromi P Murria C Macias-Hernandez N Ribera C (2007) The dark side of an island radiation: systematics and evolution of troglobitic spiders of the genus Dysdera Latreille (Araneae : Dysderidae) in the Canary Islands. Invertebrate Systematics 21: 623–660. doi: 10.1071/is07015

[B9] Bandelt HJ Forster P Rohl A (1999) Median-joining networks for inferring intraspecific phylogenies. Molecular Biology and Evolution 16: 37–48. doi: 10.1093/oxfordjournals.molbev.a026036 10331250 10.1093/oxfordjournals.molbev.a026036

[B10] Bell JR Bohan DA Shaw EM Weyman GS (2005) Ballooning dispersal using silk: world fauna, phylogenies, genetics and models. Bulletin of Entomological Research 95: 69–114. doi: 10.1079/BER2004350 15877859 10.1079/ber2004350

[B11] Bidegaray-Batista L Arnedo MA (2011) Gone with the plate: the opening of the Western Mediterranean basin drove the diversification of ground-dweller spiders. BMC Evolutionary Biology 11: . doi: 10.1186/1471-2148-11-317 10.1186/1471-2148-11-317 PMC3273451 22039781

[B12] Bloom T Binford G Alayon G Esposito L Peterson I Nishida A Loubet-Senear K Agnarsson I (2014) Discovery of two new species of eyeless spiders within a single Hispaniola cave. Journal of Arachnology 42: 148–154. doi: 10.1636/K13-84.1

[B13] Candek K Kuntner M (2015) DNA barcoding gap: reliable species identification over morphological and geographical scales. Molecular Ecology Resources 15: 268–277. doi: 10.1111/1755-0998.12304 25042335 10.1111/1755-0998.12304

[B14] Catalog WS (2015) World Spider Catalog, version 16. Natural History Museum Bern, Bern.

[B15] Cheng R-C Kuntner M (2014) Phylogeny suggests non-directional and isometric evolution of sexual size dimorphism in argiopine spiders. Evolution 68(10): 2861–2872. 25130435 10.1111/evo.12504

[B16] Cheng R-C Kuntner M (2015) Disentangling the size and shape components of sexual dimorphism. Evolutionary Biology 42: 223–234. doi: 10.1007/s11692-015-9313-z

[B17] Claramunt S Derryberry EP Remsen Jr JV Brumfield RT (2012) High dispersal ability inhibits speciation in a continental radiation of passerine birds. Proceedings of the Royal Society B-Biological Sciences 279: 1567–1574. doi: 10.1098/rspb.2011.1922 10.1098/rspb.2011.1922 PMC3282344 22090382

[B18] Clerck C (1757) Aranei Suecici, descriptionibus et figuris oeneis illustrati,ad genera subalterna redacti speciebus ultra LX determinati. Svenska Spindlar,uti sina hufvud-slagter undelte samt.. [Publ. not given], Stockholmiae.

[B19] Cosgrove J Agnarsson I Harvey M Binford G (2016) Pseudoscorpion diversity and distribution in the West Indies: sequence data confirms single island endemism for some clades, but not others. Journal of Arachnology, in press.

[B20] Crews SC Gillespie RG (2010) Molecular systematics of *Selenops* spiders (Araneae: Selenopidae) from North and Central America: implications for Caribbean biogeography. Biological Journal of the Linnean Society 101: 288–322. doi: 10.1111/j.1095-8312.2010.01494.x

[B21] Crews SC Puente-Rolón AR Rutstein E Gillespie RG (2010) A comparison of populations of island and adjacent mainland species of Caribbean *Selenops* (Araneae: Selenopidae) spiders. Molecular Phylogenetics and Evolution 54: 970–983. doi: 10.1016/j.ympev.2009.10.012 19833220 10.1016/j.ympev.2009.10.012

[B22] Darriba D Taboada GL Doallo R Posada D (2012) jModelTest 2: more models, new heuristics and parallel computing. Nature Methods 9: 772–772. doi: 10.1038/nmeth.2109 10.1038/nmeth.2109 PMC4594756 22847109

[B23] Diamond JM Gilpin ME Mayr E (1976) Species-Distance Relation for Birds of Solomon Archipelago, and Paradox of Great Speciators. Proceedings of the National Academy of Sciences of the United States of America 73: 2160–2164. doi: 10.1073/pnas.73.6.2160 16592328 10.1073/pnas.73.6.2160 PMC430470

[B24] Drummond AJ Rambaut A (2007) BEAST: Bayesian evolutionary analysis by sampling trees. BMC Evolutionary Biology 7. doi: 10.1186/1471-2148-7-214 10.1186/1471-2148-7-214 PMC2247476 17996036

[B25] Drummond AJ Suchard MA Xie D Rambaut A (2012) Bayesian Phylogenetics with BEAUti and the BEAST 1.7. Molecular Biology and Evolution 29: 1969–1973. doi: 10.1093/molbev/mss075 22367748 10.1093/molbev/mss075 PMC3408070

[B26] Dziki A Binford GJ Coddington JA Agnarsson I (2015) Spintharus flavidus in the Caribbean-a 30 million year biogeographical history and radiation of a ‘widespread species’. Peerj 3. doi: 10.7717/peerj.1422 10.7717/peerj.1422 PMC4655100 26618089

[B27] Eckert CG Samis KE Lougheed SC (2008) Genetic variation across species’ geographical ranges: the central–marginal hypothesis and beyond. Molecular Ecology 17: 1170–1188. doi: 10.1111/j.1365-294X.2007.03659.x 18302683 10.1111/j.1365-294X.2007.03659.x

[B28] Ersts PJ (2016) Geographic Distance Matrix Generator (version 1.2.3). American Museum of Natural History, Center for Biodiversity and Conservation http://biodiversityinformatics.amnh.org/open_source/gdmg [accessed on 2016-8-31]

[B29] Esposito LA Bloom T Caicedo-Quiroga L Alicea-Serrano AM Sanchez-Ruiz JA May-Collado LJ Binford GJ Agnarsson I (2015) Islands within islands: Diversification of tailless whip spiders (Amblypygi, *Phrynus*) in Caribbean caves. Molecular Phylogenetics and Evolution 93: 107–117. doi: 10.1016/j.ympev.2015.07.005 26220837 10.1016/j.ympev.2015.07.005

[B30] Fabricius JC (1775) Systema Entomologiae, sistens Insectorum classes,ordines,genera,species,adiectis,synonymis,locis descriptionibus observationibus. [Publ. not given], Flensbergi et Lipsiae.

[B31] Folmer O Black M Hoeh W Lutz R Vrijenhoek R (1994) DNA primers for amplification of mitochondrial cytochrome *c* oxidaes subunit I from diverse metazoan invertebrates. Molecular Marine Biology and Biotechnology 3: 294–299. 7881515

[B32] Gillespie RG (2005) The ecology and evolution of Hawaiian spider communities. American Scientist 93: 122–131. doi: 10.1511/2005.52.961

[B33] Gillespie RG Claridge EM Goodacre SL (2008) Biogeography of the fauna of French Polynesia: diversification within and between a series of hot spot archipelagos. Philosophical Transactions of the Royal Society B-Biological Sciences 363: 3335–3346. doi: 10.1098/rstb.2008.0124 10.1098/rstb.2008.0124 PMC2607382 18782725

[B34] Gillespie RG Roderick GK (2002) Arthropods on islands: Colonization, speciation, and conservation. Annual Review of Entomology 47: 595–632. doi: 10.1146/annurev.ento.47.091201.145244 10.1146/annurev.ento.47.091201.145244 11729086

[B35] Green P (2009) Phrap. 1.090518 ed.

[B36] Green P Ewing B (2002) PHRED. 0.020425c ed. http://phrap.org/

[B37] Hamilton JA Eckert CG (2007) Population genetic consequences of geographic disjunction: a prairie plant isolated on Great Lakes alvars. Molecular Ecology 16: 1649–1660. doi: 10.1111/j.1365-294X.2007.03241.x 17402980 10.1111/j.1365-294X.2007.03241.x

[B38] Hebert PDN Cywinska A Ball SL DeWaard JR (2003) Biological identifications through DNA barcodes. Proceedings of the Royal Society of London Series B-Biological Sciences 270: 313–321. doi: 10.1098/rspb.2002.2218 10.1098/rspb.2002.2218 PMC1691236 12614582

[B39] Heinicke MP Duellman WE Hedges SB (2007) Major Caribbean and Central American frog faunas originated by ancient oceanic dispersal. Proceedings of the National Academy of Sciences of the United States of America 104: 10092–10097. doi: 10.1073/pnas.0611051104 17548823 10.1073/pnas.0611051104 PMC1891260

[B40] Huelsenbeck JP Ronquist F (2001) MRBAYES: Bayesian inference of phylogenetic trees. Bioinformatics 17: 754–755. doi: 10.1093/bioinformatics/17.8.754 11524383 10.1093/bioinformatics/17.8.754

[B41] Iturralde-Vinent M MacPhee R (1999) Paleogeography of the Caribbean region: implications for cenozoic biogeography. Bulletin of the American Museum of Natural History 238: 1–95.

[B42] Iturralde-Vinent MA (2006) Meso-Cenozoic Caribbean Paleogeography: Implications for the Historical Biogeography of the Region. International Geology Review 48.

[B43] Jaeger P (2012) A review on the spider genus Argiope Audouin 1826 with special emphasis on broken emboli in female epigynes (Araneae: Araneidae: Argiopinae). Beitraege zur Araneologie 7: 272–331.

[B44] Jardon-Barbolla L Delgado-Valerio P Geada-Lopez G Vazquez-Lobo A Pinero D (2011) Phylogeography of Pinus subsection Australes in the Caribbean Basin. Annals of Botany 107: 229–241. doi: 10.1093/aob/mcq232 21118838 10.1093/aob/mcq232 PMC3025731

[B45] Katoh S (2013) MAFFT multiple sequence alignment software version 7: improvements in performance and usability. Molecular Biology and Evolution 30: 772–780. doi: 10.1093/molbev/mst010 23329690 10.1093/molbev/mst010 PMC3603318

[B46] Kimura M (1980) A simple method for estimating evolutionary rate of base substitutions through comparative studies of nucleotide sequences. Journal of Molecular Evolution 16: 111–120. doi: 10.1007/BF01731581 7463489 10.1007/BF01731581

[B47] Kuntner M Agnarsson I (2011a) Biogeography and diversification of hermit spiders on Indian Ocean islands (Nephilidae: *Nephilengys*). Molecular Phylogenetics and Evolution 59: 477–488. doi: 10.1016/j.ympev.2011.02.002 21316478 10.1016/j.ympev.2011.02.002

[B48] Kuntner M Agnarsson I (2011b) Phylogeography of a successful aerial disperser: the golden orb spider *Nephila* on Indian Ocean islands. BMC Evolutionary Biology 11: . doi: 10.1186/14-71-2148-11-119 10.1186/1471-2148-11-119 PMC3098804 21554687

[B49] Levi HW (1968) The spider genera *Gea* and *Argiope* in America (Araneae: Araneidae). Harvard University, Cambridge.

[B50] Levi HW (1983) The orb-weaver genera *Argiope*, *Gea* and *Neogea* from the western Pacific region (Araneae: Argiopinae, Araneidae). Bulletin of the Museum of Comparative Zoology 150: 247–338.

[B51] Levi HW (2004) Comments and new records for the American genera Gea and Argiope with the description of a new species (Araneae: Araneidae). Bulletin of the Museum of Comparative Zoology 158: 47–65. doi: 10.3099/0027-4100(2004)158[47:canrft]2.0.co;2

[B52] Librado P Rozas J (2009) DnaSP v5: A software for comprehensive analysis of DNA polymorphism data. Bioinformatics 25: 1451–1452. doi: 10.1093/bioinformatics/btp187 19346325 10.1093/bioinformatics/btp187

[B53] Maddison DR Maddison WP (2011a) Chromaseq: a Mesquite module for analyzing sequence chromatograms. version 1.0. http://mesquiteproject.org/packages/chromaseq

[B54] Maddison W Maddison D (2011b) Mesquite: a modular system for evolutionary analysis. 2.75 (build 566) ed. http://mesquiteproject.org

[B55] McHugh A Yablonsky C Binford G Agnarsson I (2014) Molecular phylogenetics of Caribbean Micrathena (Araneae : Araneidae) suggests multiple colonisation events and single island endemism. Invertebrate Systematics 28: 337–349. doi: 10.1071/is13051

[B56] Pons J Barraclough TG Gomez-Zurita J Cardoso A Duran DP Hazell S Kamoun S Sumlin WD Vogler AP (2006) Sequence-based species delimitation for the DNA taxonomy of undescribed insects. Systematic Biology 55: 595–609. doi: 10.1080/10635150600852011 16967577 10.1080/10635150600852011

[B57] Posada D Buckley TR (2004) Model selection and model averaging in phylogenetics: advantages of the AIC and Bayesian approaches over likelihood ratio tests. Systematic Biology 53: 793–808. doi: 10.1080/10635150490522304 15545256 10.1080/10635150490522304

[B58] R Core Team (2014) R: A language and environment for statistical computing. R Foundation for Statistical Computing, Vienna.

[B59] Ricklefs R Bermingham E (2008) The West Indies as a laboratory of biogeography and evolution. Philosophical Transactions of the Royal Society B-Biological Sciences 363: 2393–2413. doi: 10.1098/rstb.2007.2068 10.1098/rstb.2007.2068 PMC2606802 17446164

[B60] Tamura K Stecher G Peterson D Filipski A Kumar S (2013) MEGA6: Molecular Evolutionary Genetics Analysis Version 6.0. Molecular Biology and Evolution 30: 2725–2729. doi: 10.1093/molbev/mst197 24132122 10.1093/molbev/mst197 PMC3840312

[B61] Warren BH Simberloff D Ricklefs RE Aguilee R Condamine FL Gravel D Morlon H Mouquet N Rosindell J Casquet J Conti E Cornuault J Maria Fernandez-Palacios J Hengl T Norder SJ Rijsdijk KF Sanmartin I Strasberg D Triantis KA Valente LM Whittaker RJ Gillespie RG Emerson BC Thebaud C (2015) Islands as model systems in ecology and evolution: prospects fifty years after MacArthur-Wilson. Ecology Letters 18: 200–217. doi: 10.1111/ele.12398 25560682 10.1111/ele.12398

[B62] Weeks BC Claramunt S (2014) Dispersal has inhibited avian diversification in Australasian archipelagoes. Proceedings of the Royal Society B-Biological Sciences 281. doi: 10.1098/rspb.2014.1257 10.1098/rspb.2014.1257 PMC4132686 25100701

[B63] Zhang J-X Maddison WP (2012) New euophryine jumping spiders from the Dominican Republic and Puerto Rico (Araneae: Salticidae: Euophryinae). Zootaxa 3476: 1–54.

