# Supplementary figures and images for: Phylogeography of a good Caribbean disperser: Argiope argentata (Araneae, Araneidae) and a new ‘cryptic’ species from Cuba
Source: Zookeys. 2016 Oct 19;(625):25–44. doi: 10.3897/zookeys.625.8729 (PMC5096361; doi:10.3897/zookeys.625.8729)

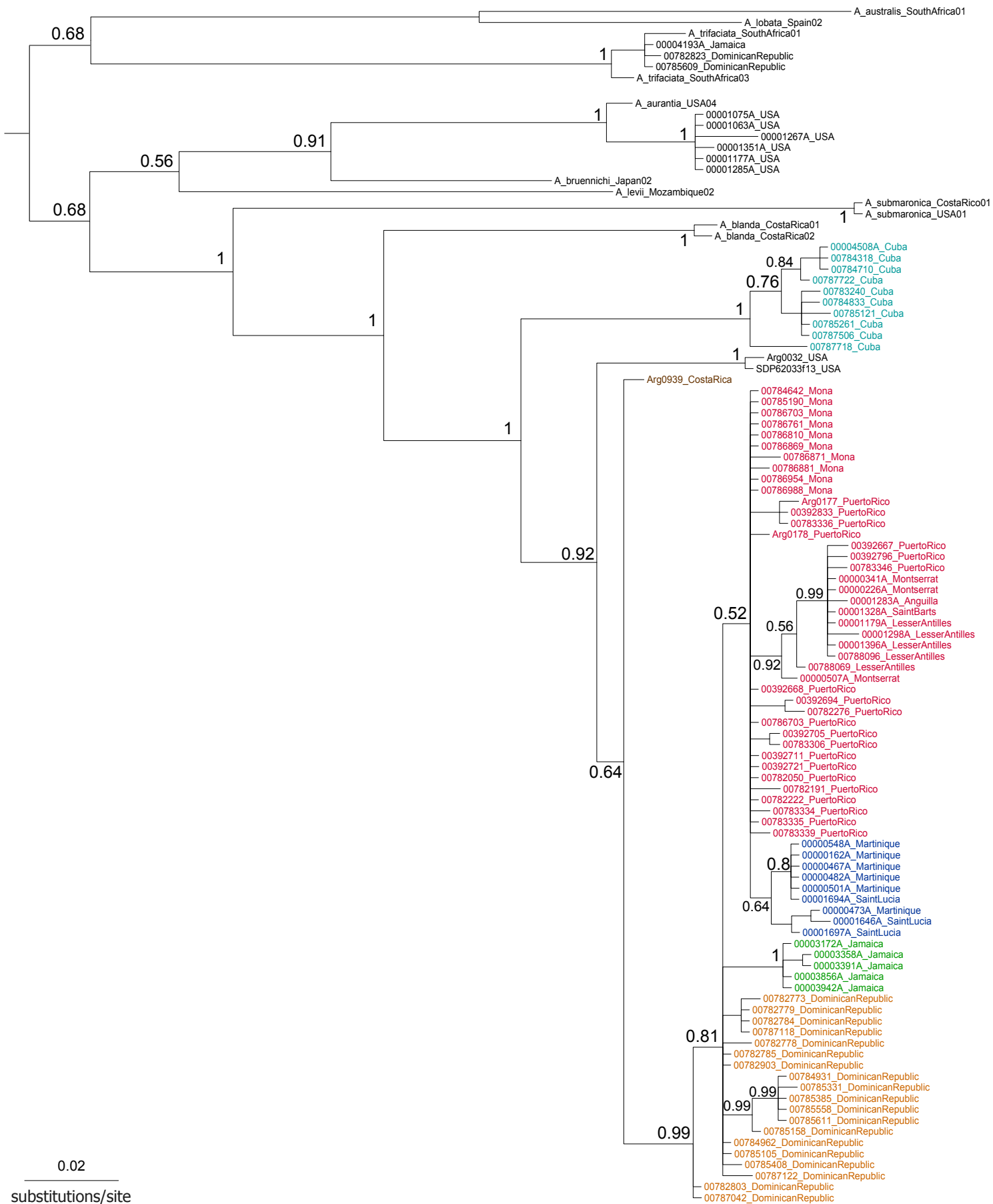

Supplement: Supplementary material 1 — Figure S1 [file zookeys-625-025-s001.pdf]

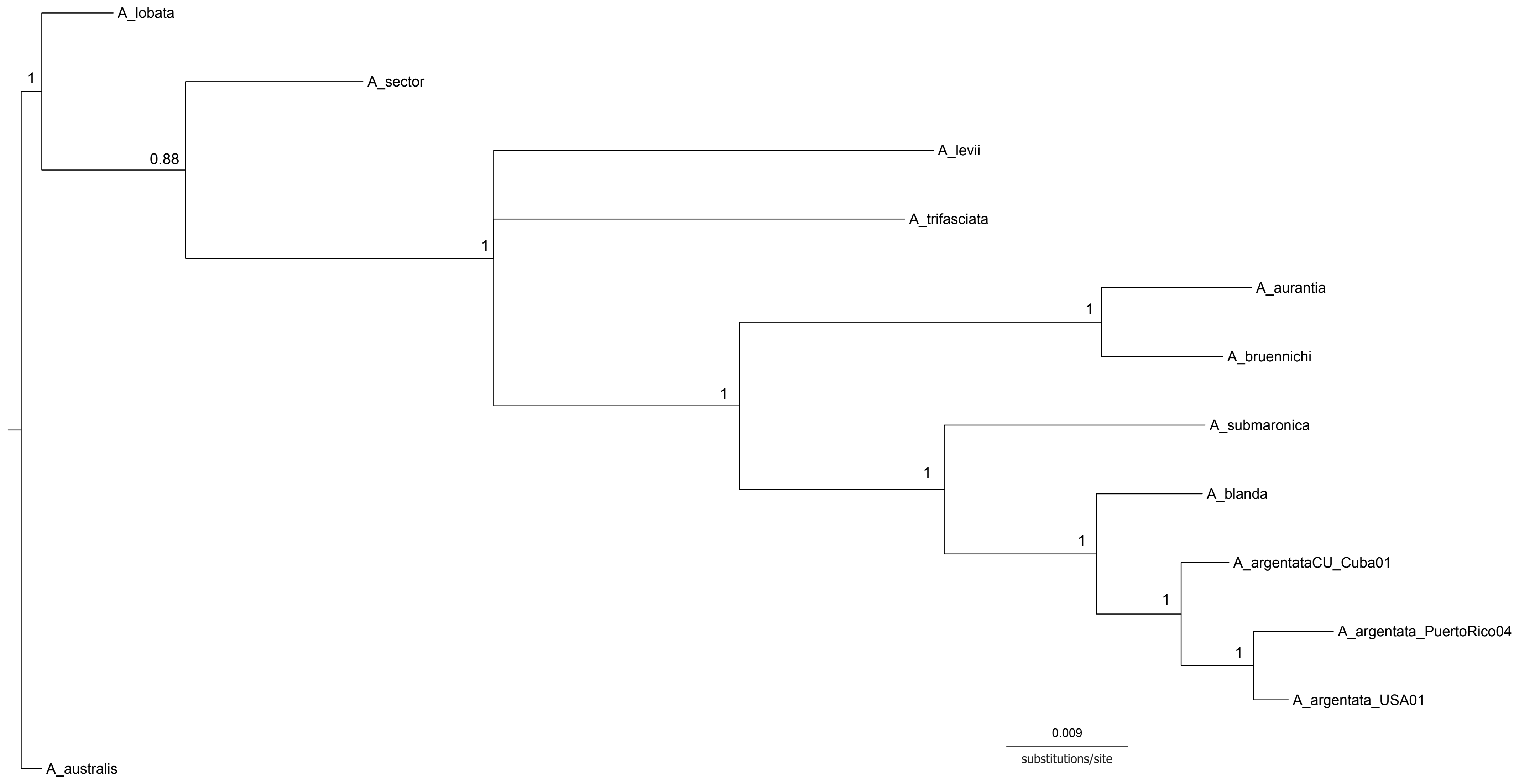

Supplement: Supplementary material 2 — Figure S2 [file zookeys-625-025-s002.pdf]

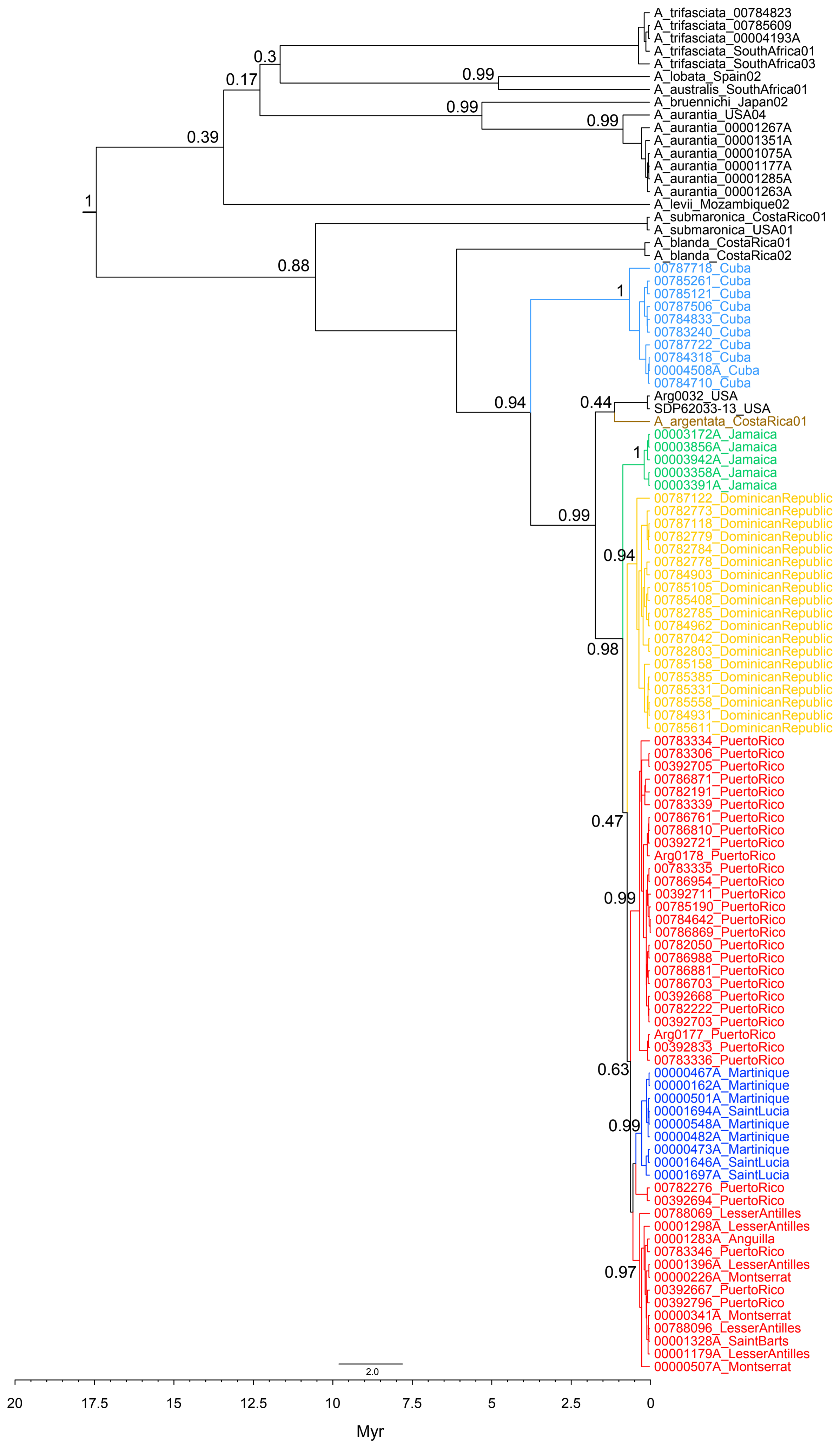

Supplement: Supplementary material 3 — Figure S3 [file zookeys-625-025-s003.pdf]
